# Interleukin-17-producing decidual CD4+ T cells are not deleterious for human pregnancy when they also produce interleukin-4

**DOI:** 10.1186/s12948-016-0039-y

**Published:** 2016-01-21

**Authors:** Letizia Lombardelli, Federica Logiodice, Maryse Aguerre-Girr, Ornela Kullolli, Herman Haller, Ysabel Casart, Alain Berrebi, Fatima-Ezzahra L’Faqihi-Olive, Valérie Duplan, Sergio Romagnani, Enrico Maggi, Daniel Rukavina, Philippe Le Bouteiller, Marie-Pierre Piccinni

**Affiliations:** Department of Experimental and Clinical Medicine and DENOTHE Excellence Center, University of Florence, Largo Brambilla 3, 50134 Florence, Italy; INSERM UMR1043, CNRS UMR5282, Centre de Physiopathologie Toulouse-Purpan, Université de Toulouse III, 31024 Toulouse, France; Gynécologie-Obstétrique, Hôpital Paule de Viguier, Toulouse, France; Department of Physiology and Immunology, Medical Faculty, University of Rijeka, 51000 Rijeka, Croatia; Department of Gynecology and Obstetrics, Medical Faculty, University of Rijeka, 51000 Rijeka, Croatia

**Keywords:** Th17, Pregnancy, IL-17, IL-4, Spontaneous abortion, Ectopic pregnancy

## Abstract

**Background:**

Trophoblast expressing paternal HLA-C antigens resemble a semiallograft, and could be rejected by maternal CD4+ T lymphocytes. We examined the possible role in human pregnancy of Th17 cells, known to be involved in allograft rejection and reported for this reason to be responsible for miscarriages. We also studied Th17/Th1 and Th17/Th2 cells never investigated before. We defined for the first time the role of different Th17 subpopulations at the embryo implantation site and the role of HLA-G5, produced by the trophoblast/embryo, on Th17 cell differentiation.

**Methods:**

Cytokine production by CD4+ purified T cell and T clones from decidua of normal pregnancy, unexplained recurrent abortion, and ectopic pregnancy at both embryo implantation site and distant from that site were analyzed for protein and mRNA production. Antigen-specific T cell lines were derived in the presence and in the absence of HLA-G5.

**Results:**

We found an associated spontaneous production of IL-17A, IL-17F and IL-4 along with expression of CD161, CCR8 and CCR4 (Th2- and Th17-type markers) in fresh decidua CD4+ T cells during successful pregnancy. There was a prevalence of Th17/Th2 cells (producing IL-17A, IL-17F, IL-22 and IL-4) in the decidua of successful pregnancy, but the exclusive presence of Th17 (producing IL-17A, IL-17F, IL-22) and Th17/Th1 (producing IL-17A, IL-17F, IL-22 and IFN-γ) cells was found in the decidua of unexplained recurrent abortion. More importantly, we observed that Th17/Th2 cells were exclusively present at the embryo implantation site during tubal ectopic pregnancy, and that IL-4, GATA-3, IL-17A, ROR-C mRNA levels increased in tubal biopsies taken from embryo implantation sites, whereas Th17, Th17/Th1 and Th1 cells are exclusively present apart from implantation sites. Moreover, soluble HLA-G5 mediates the development of Th17/Th2 cells by increasing IL-4, IL-17A and IL-17F protein and mRNA production of CD4+ T helper cells.

**Conclusion:**

No pathogenic role of decidual Th17 cells during pregnancy was observed. Indeed, a beneficial role for these cells was observed when they also produced IL-4. HLA-G5 could be the key feature of the uterine microenvironment responsible for the development of Th17/Th2 cells, which seem to be crucial for successful embryo implantation.

**Electronic supplementary material:**

The online version of this article (doi:10.1186/s12948-016-0039-y) contains supplementary material, which is available to authorized users.

## Background

The conceptus, because of the presence of paternal classical MHC class I antigens (HLA-C) [1], is thought to resemble a semiallograft [2]. Paternal antigens expressed by trophoblast could be processed and presented, together with self MHC class II, to the specific maternal CD4+ T helper cells by maternal antigen presenting cells (APCs). Consequently, the activated maternal effector CD4+ T helper cells could release various cytokines.

On the basis of the cytokines produced, the human effector CD4+ T helper cells have been classified as T helper (Th)1, (Th)2 and, more recently, as (Th17) cells [3, 4]. Indeed, CD4+ Th1 cells, which produce interleukin (IL)-2, tumor necrosis factor (TNF)-α and interferon (IFN)-γ are the main effectors of phagocyte-mediated host defense, which is highly protective against infections sustained by intracellular parasites. On the other hand, CD4+ Th2 cells, which are mainly responsible for phagocyte-independent host defense against extracellular parasites, including nematodes, produce IL-5 (promoting the growth and the differentiation of eosinophils) and IL-4 (which together with IL-13 stimulates IgE and IgG1 antibody production and inhibits several macrophage functions) [3]. An additional subset of CD4+ T helper cells identified as Th17 produces IL-17A, IL17F, IL-21, IL-26 and IL-22 [4–6]. There is an increasing body of evidence showing that Th17 cells constitute a novel Th cell lineage distinct from Th1 and Th2 cells [4]. It has been reported that the transcription factor retinoic-acid-related orphan receptor (ROR)-C is important for the generation of Th17 cells in vitro and in vivo [7]. Human Th17 cells appear to be quite different from mouse Th17 cells in that TGF-β1 and IL-6 are not required for generation of Th17 cells. To date, the most effective cytokines to enhance the generation or expansion of human Th17 cells are IL-1β and IL-23 [8, 9]. In addition, cytokines such as IL-23 and IL-21 promote the generation or proliferation of Th17 cells, whereas others such as IFN-γ, IL-4, and IL-27, seem to suppress their generation [5, 6, 8, 10, 11]. The major role of Th17 is the protection against extracellular bacteria by activating epithelial cells, macrophages, fibroblasts and endothelial cells, which produce chemokines and cytokines responsible for granulocyte recruitment, which contributes to chronic tissue inflammation. The pathogenic role of Th17 cells has been suggested in several murine models of chronic inflammatory disorders, such as experimental autoimmune encephalomyelitis (EAE) [11], collagen-induced arthritis [9], and bowel inflammatory disorders [12].

Importantly for pregnancy, Th1-type and Th17-type T helper cells seem to play a role in acute allograft rejection [13–18], whereas Th2 T helper cells [15] and CD4+ CD25+Foxp3+ T reg cells act to enhance allograft tolerance [19]. Although the mechanisms of action of Th1- and Th2-type cytokines produced by maternal CD4+ T helper lymphocytes present at the fetomaternal interface are defined with respect to conceptus tolerance/pregnancy maintenance or are conceptus rejection/failure of pregnancy [20–24], the mechanisms of action of CD4+CD25+Foxp3+ T reg cells present during human pregnancy are not so clear [25]. It was suggested in human pregnancy that T reg cells could inhibit the majority of human T cells that spontaneously proliferate and produce IFN-γ [26]. Kallikourdis et al. [27] reported that paternal alloantigen enhanced the accumulation of CCR5+ effector Treg cells in the murine pregnant uterus. Shima et al. [28] also reported that paternal antigen-specific Ki67+ proliferating Treg cells expressed CCR5 on their surface. These findings suggest that CCR5+ proliferating T cells might induce paternal antigen-specific tolerance in humans. However, recently Inada et al. [29] reported that the frequencies of CCR5^+^ Treg cells did not change in miscarriage. Unexpectedly, this study showed that the frequencies of CCR5^+^ Treg cells and Ki67+ Treg cells are similar in cases of normal pregnancy, miscarriage with a normal embryo, and miscarriage with an abnormal embryo, suggesting that decidual T reg specific for paternal antigens could be not responsible for the success of pregnancy by inducing fetal allograft tolerance. Accordingly, in mice it seems that T reg cells are activated in the uterine lymph nodes in response to semen and seminal antigens, not spermatozoa antigens and they home back to the uterus where they prepare the endometrium for implantation [30]. Presumably, the T reg cells in part act to dampen uterine inflammation, which is induced by semen and is independent of placental antigen exposure.

In the transplant setting, it was widely believed that allograft rejection is predominantly a Th1-mediated immune response, and that Th2-type cytokines inhibiting the Th1 responses improve allograft tolerance. It has been shown that a new T helper cell subpopulation, known as Th17 has a role in early stage allograft acute rejection [13, 14]. Therefore, the role of Th17 cells in conceptus rejection pregnancy failure has been investigated. Early evidence suggests that excessive Th17 activity may promote miscarriage. The number of Th17 CCR6+ cells is increased in the peripheral blood and decidua of patients with unexplained recurrent miscarriage compared to healthy control subjects, whereas T reg cells are decreased [31]. Accordingly, it has been shown that IL-27, a key regulator of T cell responses suppressing in particular Th17 cells, is lower in deciduas of patients with unexplained recurrent abortion compared to spontaneous abortion and controls subjects [32]. An accumulation of Th17 cells has also been found in the decidua of spontaneous abortion cases [33], and the number of decidual IL-17+ cells in inevitable abortion cases involving active genital bleeding was significantly higher than that in normal pregnancy. However, it seemed that there were no significant differences in the number of decidual IL-17+ cells between missed abortion cases without genital bleeding and normal pregnancy subjects. These last results suggest that IL-17+ cells might be involved in the induction of inflammation in the late but not the early stages of abortion [33]. Thus, the role of Th17 cells in the induction of spontaneous abortion remains unclear.

It has been reported that virtually all human memory Th17 cells are contained within the CD161+ fraction of circulating and tissue-infiltrating CD4+ T cells [10, 12]. Interestingly, human Th17 cells express molecules distinct from Th1 cells, such as IL-23R and RORC, but other molecules are shared with Th1 cells, such as the T-box 21 (TBX21) and IL-12Rβ2. Furthermore, a portion of human IL-17A-producing cells were found to also produce IFN-γ (they are named Th17/Th1) and both Th17 and Th17/Th1 exhibit plasticity towards Th1 cells in response to IL-12 produced in by APCs [11]. This plasticity of Th17 cells to Th1 cells has recently been observed even in mice, where it was found to be related to the activity of IL-12, or the prolonged exposure to IL-23 of Th17 cells [34]. Thus, naive CD4+ CD161+ T cells that behave as precursors of Th17 cells [10] could differentiate into Th17, Th17/Th1 and finally into Th1 cells, in response to cytokines present in the microenvironment of CD4+ T cells. The association of IL-17 and IL-4 production by CD4+ T helper cells has also been observed recently in allergic disorders [35]. A small proportion of CCR6(+)CD161(+)CD4(+) T cell clones showed the ability to produce both IL-17A and IL-4 (Th17/Th2). Th17/Th2 clones also produced IL-5, IL-13, IL-21, and IL-22 and displayed the ability to induce the in vitro secretion of IgE. Very few Th17/Th2 cells were found among circulating CD4(+) T cells from normal subjects (0.04 %), but their proportions were significantly increased in the circulation of patients with chronic asthma (1.3 %). Th17/Th2 cells could not be derived from naive umbilical cord blood CD4(+) T cells under any experimental condition. However, when circulating memory CCR6(+)CD161(+)CD4(+) T cells were cloned under appropriate polarizing conditions, Th17/Th2 clones originated in the presence of IL-4, suggesting that an IL-4-rich microenvironment may induce the shifting of memory Th17 cells into Th17/Th2 cells.

The aims of our study were (1) to define the role of Th17 cells in pregnancy, in particular to investigate if, as was reported by Wang et al. [31], these cells are responsible for miscarriages; (2) to define to which Th17-type subpopulation (Th17/Th1 or Th17/Th2 cells) the decidual Th17 cells belong and to ascertain whether one of these two Th17-type profiles is preferentially associated with pregnancy failure or successful pregnancy; (3) to find a putative soluble factor present at the fetomaternal interface responsible, at least in part, for the differentiation of decidual CD4+ T cells into Th17/Th2 cells.

## Methods

### Subjects

Among the 36 pregnant women studied (Table 1), 30 pregnant women, agreed to participate to the study at Hôpital Paule de Viguier, Toulouse, France. An additional six pregnant women suffering from ectopic pregnancy agreed to participate in the study at Hospital of Rijeka, Croatia. All subjects received verbal and written information about the aim and the design of the research, and all pregnant women signed the informed consent. The study was approved by local ethics committees of Hôpital Paule de Viguier, Toulouse, and by the Medical Faculty, Rijeka. Twenty-six pregnant women with normal gestation and no spontaneous abortion in their past history (Table 1), had requested elective termination. Four women had histories of at least 7 ± 3 (range 4–10) prior first-trimester spontaneous abortions, which could not been explained on the basis of conventional criteria (normal parental chromosomes, hysterosalpengography and hysteroscopy, endometrial biopsy, hormonal analysis including FSH, LH, estradiol, testosterone, cervical cultures for the presence of ureaplasma, mycoplasma and chlamydia, lupus anticoagulant, anti-phospholipid antibodies, thyroid function tests). Specimens of deciduae and peripheral blood were obtained at the time of spontaneous abortion (at 8–11 weeks of pregnancy with normal karyotype of trophoblast). All the women were in excellent health at that time, had no history of atopy or allergy and were taking no medication. Trophoblast-invaded tubal mucosa at the implantation site and tubal mucosa distant from the implantation site was obtained from 6 women (with no spontaneous abortion in past history) whose ectopic pregnancies were terminated by surgical removal as a result of threatened tubal rupture. The mean age and the gestational age values of the three groups of patients (normal pregnancy, unexplained recurrent abortion and ectopic pregnancy) were not statistically different (Table 1).

**Table 1:** Clinical data from patients (unexplained recurrent abortion, ectopic, pregnancy and controls)

	Normal pregnancy	Unexplained recurrent abortion	Ectopic pregnancy
*n*	26	4	6
Age (years)	28 ± 2 (25–35)	29 ± 0.9 (28–30)	32 ± 3 (29–36)
No. of Sp-ab^a^	0	7 ± 3 (4–10)	0
Gestational age (weeks)	9 ± 1 (8–12)	9 ± 1 (8–11)	8 ± 1 (6–9)

Data are expressed as mean ± SD (range)

^a^Number of patients with spontaneous abortion in past history, excluding the abortion cases discussed in this study

### Isolation of purified CD4+ T cells from peripheral blood and *decidua basalis* of early pregnant women

Samples of *decidua basalis* were obtained from healthy pregnant women undergoing vaginal elective termination of pregnancy (8–12 weeks of gestation with normal karyotype of trophoblast). Decidual mononuclear cells were isolated from the *decidua basalis* by collagenase digestion and gradient centrifugation as previously described [36]. Decidual CD4+ T cells were purified from non adherent cells using MACS CD4 isolation kit (positive selection, Miltenyi Biotec, Bergisch Gladbach, Germany). Purity was routinely >98 %. Peripheral blood (PB) cells from the same pregnant women were obtained as described [37]. Peripheral blood-CD4+ T cells were purified by using MACS CD4 isolation kit (positive selection, Miltenyi Biotec, Bergisch Gladbach, Germany). Purity was >99 %.

### Flow cytometry

Freshly isolated decidual CD4+ T and Peripheral blood-CD4+ T cells were stained simultaneously with CD3-PE-Cy7, CD4-pacific blue, CD161-APC (BD Biosciences, Franklin Lakes, New Jersey) and either CCR3-FITC (Miltenyi Biotec, Bergisch Gladbach, Germany), IL-23R-PerCP, CCR4-mouse PE, CCR8-rat PE, CCR6-PE, CCR8-rat-PE, CXCR3 mouse-PE (R&D systems, Minneapolis, MN), or CRTH2 rat-PE (Myltenyi Biotech, Bergisch Gladbach, Germany) mAbs or their respective isotype controls: IgG1 mouse PE-Cy7, IgG1 mouse-pacific blue, IgG1 mouse APC, IgG2a rat-FITC, IgG2b mouse-PerCP, IgG1 mouse-PE, IgG2a rat-PE (BD Biosciences, Franklin Lakes, New Jersey), IgG2b-mouse PE, IgG2b-rat PE (R&D systems, Minneapolis, MN). Stained cells were acquired on a BD Biosciences LSR II flow cytometer (BD Biosciences, Franklin Lakes, New Jersey) (Data were analyzed with BD Biosciences FACSDiva software version 6.2.

### Generation of CD4+ T-cell clones from peripheral blood, decidual biopsies of normal pregnancy unexplained recurrent abortion, and from Fallopian tube biopsies of ectopic pregnancy

Specimen of deciduae (separated from villus with normal karyotype) and of Fallopian tubes, were washed twice in PBS (pH 7.2) and then disrupted in small fragments (2–3 mm in diameter). Short-term T-cell lines were generated by culturing single fragments for one week in 24-well plates (Costar, Cambridge, Massachusetts) in 2 ml RPMI 1640 supplemented with 2 mM l-glutamine, 20 mM l-mercaptoethanol, 10 % FCS (complete medium) (Hyclone Laboratories, Logan, Utah) and IL-2 (Eurocitus, Milan, Italy) (20 U/ml). T-cell clones were then generated from short-term cultures of decidual and tubal T cells derived in the presence of IL-2, as well as from PBMC obtained from the same donors, using to a method described elsewhere [22].

### Induction of cytokine production by T-cell clones

To induce cytokine production, 10^6^ T-cell blasts from each T-cell clone were cultured in the presence of PMA (20 ng/ml; Sigma, St. Louis, MO) plus monoclonal antibody against CD3 (100 ng/ml; Ortho Pharmaceuticals, Raritan, New Jersey). After 36 h, culture supernatants were collected, filtered, and stored in aliquots at −70°.

### Determination of cytokine concentrations in supernatants with bead-based multiplex immunoassays

The quantitative determination of the following cytokines: IL-4, IL-5, IL-13, IL-17A, I and FN-γ was performed by a bead-based multiplex immunoassay (Biorad Laboratories, Hercules, CA, USA) and IL-17F and IL-22 (Millipore, Billerica, Massachusetts) a Bioplex 200 system (Biorad Laboratories, Hercules, CA, USA), as previously described [38]. In brief, supernatant was added to antibody-conjugated beads directed against the cytokines listed above in a 96-well filter plate. After a 30-min incubation, the plate was washed and biotinylated anti-cytokine antibody solution was added before another 30-min incubation. The plate was then washed and streptavidin-conjugated PE was added. After a final wash, each well was suspended with assay buffer and analyzed with the Bioplex 200 system. Standard curves were derived from various concentrations of the different cytokine standards following the same protocol as the supernatant samples. The concentration of each cytokine (pg/ml) in each T cell clone supernatant was calculated thanks to the Bioplex200 software.

### Cytokine production and mRNA expression of antigen-specific T cell lines in the absence or presence of HLA-G5

Recombinant HLA-G5 protein was purified from specific transfectant cell culture supernatants as previously described [39]. Streptokinase (SK)-specific T cell lines were generated from 5 donors as described elsewhere [40]. Briefly, 10^6^ PBMC in 2 ml of complete medium were stimulated for 5 days with the SK antigen (1000 U/ml) in the absence or presence of either HLA-G5 (1 µg/ml) or recombinant human IL-4 (RD System, 200 pg/ml, Minneapolis) and IL-12 (RD System, 5000 pg/ml) as controls of cytokine modulation. Human IL-2 (Eurocetus, Milan) at 20 U/ml was then added and cultures continued for an additional 9 days. Viable T blasts were tested for their antigen specificity as follows: T cell lines, 2 × 10^4^ T blasts were seeded in microplates and co-cultured for 48 h with irradiated (9000 rad) autologous PBMC (5 × 10^4^) in the presence of medium alone or SK (1000 U/ml). After a 16-h pulse with 0.5 µCi ^3^H-TdR (Amersham), cultures were harvested and radioactivity measured by liquid scintillation. The phenotype distribution of SK-specific T cells was assessed by flow cytometry analysis: the T cell lines were CD4+ cells. To induce the cytokine production by T cell lines, 10^6^ T blasts from each were cultured in the presence of PMA (Sigma, 20 ng/ml, St. Louis, MO) plus anti-CD3 mAb (BD Bioscience, 100 ng/ml, Franklin Lakes, New Jersey). After 36 h, culture supernatants were collected and stored at −80 °C. IL-4, IL-17A, IFN-γ and IL-17F were quantified by bead-based multiplex assay. Values of the cytokine content 5 SD over those of control supernatants obtained by stimulation of irradiated feeder cells alone were considered as an effective secretion.

### Quantification by real-time quantitative RT-PCR of IL-4, IL-17A, IL-17F, IL-23R, IFN-γ RORC, and GATA3 mRNA

Total RNA was extracted from freshly isolated PB and decidual CD4+ T cells, Fallopian tube biopsies and streptokinase (SK)-specific T cell lines by using Trizol (Invitrogen, Carlsbad, CA) and treated with DNase I (Qiagen, Venlo, NL). First strand cDNA was prepared from 1–5 µg of each RNA sample using Superscript II Reverse transcriptase according to the manufacturer’s instructions (Invitrogen). Total RNA was extracted with RNAsy Kit and treated with DNase I (Qiagen, Venlo, NL), and cDNA was synthetized by using TaqMan Reverse Transcription Reagents (Applied Biosystem, Warrington, UK). RT-PCR was then performed by using TaqMan methodology, as described [40]. Quantitative analysis of IL-4, IL-17A, IL-17F, IL-23R, IFN-γ, RORC, GATA 3 and β-actin was performed by using assay on Demand (Applied Biosystem, Warrington, UK). β-actin was used for normalization.

### Statistics

Statistical analyses were performed using SSPS software (SPSS, Inc, Evanston, IL). Due to non parametric distribution, all comparisons between cytokine concentrations in basal and stimulated conditions were performed by Wilcoxon test. Th subpopulations percentages were analyzed by Chi-square test. A p value of <0.05 was considered statistically significant.

## Results

### Associated production of IL-17 and IL-4 and expression of Th17 and Th2-type molecules by decidual CD3+CD4+ T cells in successful pregnancy

**Fig. 1:**
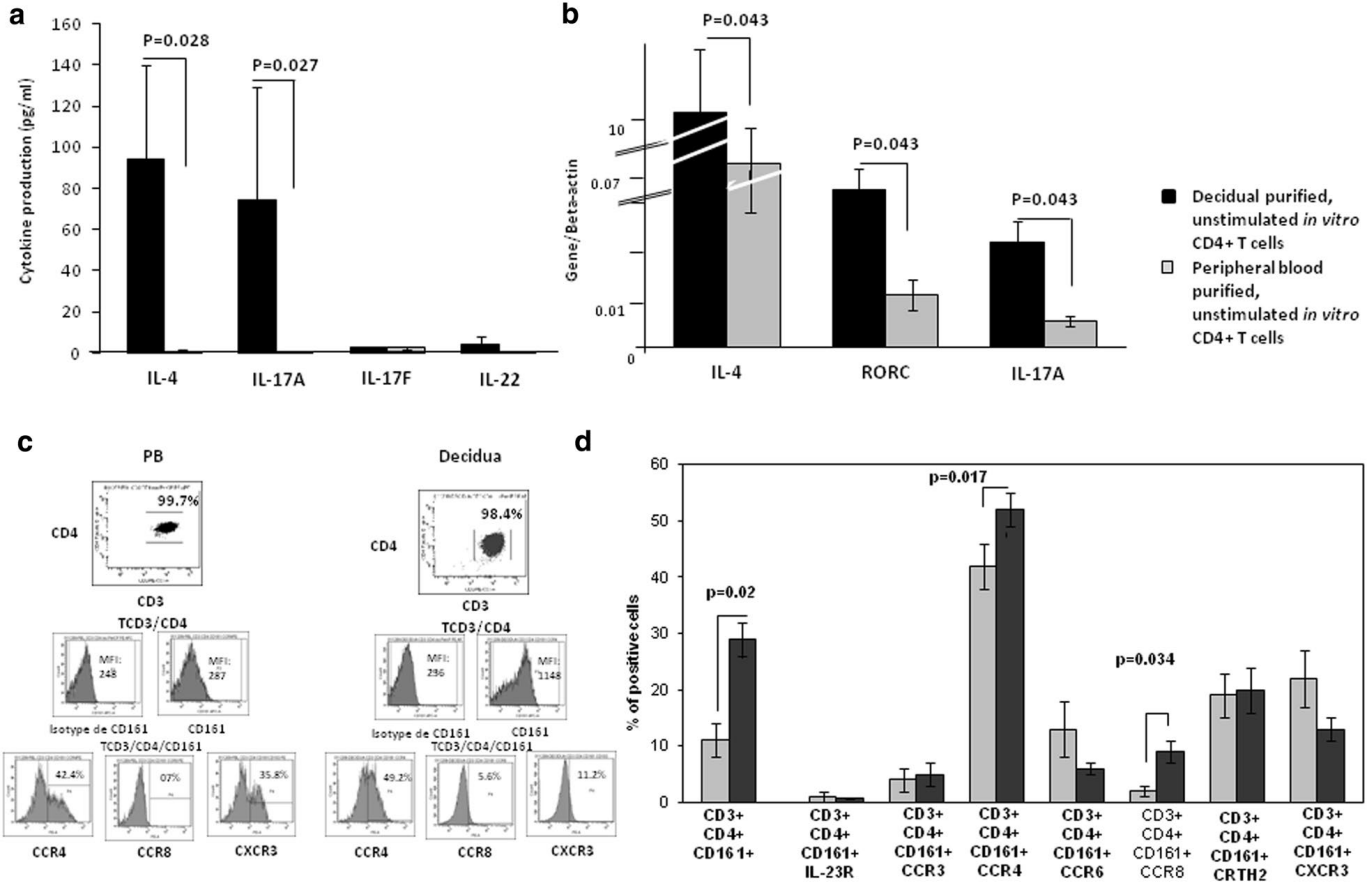
Associated spontaneous production of both IL-17A and IL-4 and expression of CD161, CCR8 and CCR4 by fresh decidua CD3+CD4+ T cells in successful pregnancy. **a** Unstimulated decidual and PB CD4+ T cells purified from the same 9 pregnant women were cultured for 24 h and IL-4, IL-17A, IL-17F and IL-22 production (mean ± SEM) was measured. **b** RT-PCR of unstimulated decidual and PB CD4+ T cells purified from the same 3 pregnant women for IL-17A, IL-4 and RORC was performed. **c**, **d** Th1 cells express CXCR3, whereas Th2 cells express CCR4, CCR8 and CRTH2 and Th17 cells express CD161, IL-23 receptor (IL-23R) CCR6 and CCR4. The associated expression of molecules expressed by Th17 cells and by Th1 and Th2 cells in fresh unstimulated CD4+ T cells purified from decidua and PB of the same pregnant women were analyzed by 4-color (CD3, CD4, CD161 and CCR4 or CCR8 or CXCR3) flow cytometry. **c** is a representative experiment and **d** data are represented as mean ± SEM. For **a**, **b** and **d** the statistical analysis was performed with Wilcoxon test

Unstimulated decidual and peripheral blood CD4+CD3+ T cells purified from the same 9 pregnant women were cultured for 24 h and IL-4, IL-17A, IL-17F and IL-22 production was measured in the corresponding cell culture supernatants (Fig. 1a). A significant increase of IL-4 and IL-17A release (p = 0.028 and p = 0.027, respectively) was observed in the culture supernatants of freshly isolated, unstimulated in vitro decidual CD4+ T cells compared to those of peripheral blood CD4+ cells from the same pregnant women (Fig. 1a). By contrast, no significant increase of IL-17F and IL-22 production was detected in the same culture supernatants of decidual CD4+ T cells compared to those of peripheral blood CD4+ cells from the same pregnant women. These data were confirmed by the increased levels of mRNA for IL-4, IL-17A and RORC (transcriptional factor of Th17 cells) expressed by freshly isolated, unstimulated decidual CD4+ T cells compared to those of peripheral blood CD4+ cells from the same 3 pregnant women (Fig. 1b). These results show a spontaneous, associated production of both IL-17A and IL-4 by fresh decidual CD3+CD4+ T cells during normal pregnancy.

Th1 cells express CXCR3, whereas Th2 cells express CCR4, CCR8 and CRTH2 [41–43]. Recent reports [10, 12] suggest that Th17 cells express CD161, IL-23 receptor (IL-23R) CCR6 and CCR4, as Th2 cells, but not CXCR3 in human adult peripheral blood or CCR6 in human decidua. We compared the associated expression of molecules expressed by Th17 cells and molecules expressed by Th1 and Th2 cells by fresh unstimulated CD3+CD4+ T cells purified from decidua and peripheral blood of the same pregnant women (Fig. 1c, d). Using multicolor flow cytometry analysis, we found that the percentage of CD3+CD4+ cells expressing CD161 (p = 0.027) and the mean fluorescence intensity (MFI) of CD161 were increased in the decidua compared to peripheral blood of the same 8 pregnant women (Fig. 1c). More importantly, this increased expression of CD161 by decidua CD3+CD4+ cells is associated with the increased expression of CCR4 and CCR8 by decidual CD3 + CD4+ cells compared to the peripheral blood CD3+CD4+ cells (Fig. 1d). These results indicate that decidual CD4+ T cells spontaneously expressed on their cell membrane molecules that characterize Th17 and Th2 cells.

### Increased IL-4, IL-17A, IL-17F and IL-22 production by decidual CD4+ T cell clones in successful pregnancy

172 and 55 CD4+ T cell clones were respectively generated from decidual biopsies and peripheral blood obtained from 4 pregnant women (with normal pregnancy) who voluntarily underwent an elective termination of pregnancy. IL-4, IL-17A, IL-17F, IL-22 and IFN-γ were measured in the supernatant of the CD4+ T cell clones by multiplex bead-based assay.

**Fig. 2:**
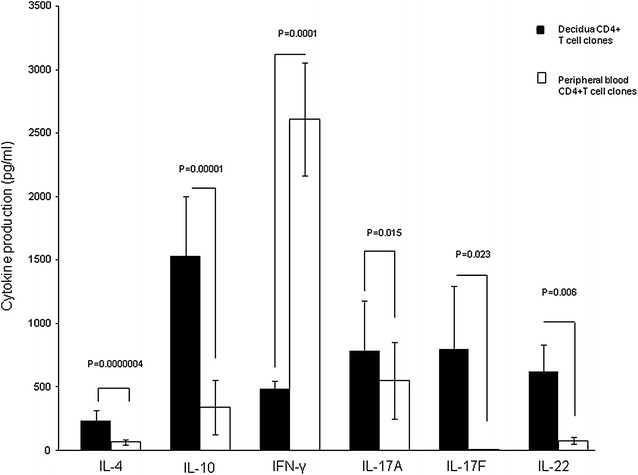
Significant increase of both IL-4 and IL-17A production by CD4+ T cell clones in decidua compared to PB in successful pregnancy. 172 and 55 CD4+ T cell clones were respectively generated from decidual biopsies and peripheral blood obtained from 4 pregnant women who underwent an elective termination of pregnancy. IL-4, IL-17A, IL-17F, IL-22, and IFN-γ (mean ± SEM) were measured in the supernatant by multiplex bead-based assay. The statistical analysis was performed with Wilcoxon test

In normal pregnancy, decidua CD4+ T cell clones produce higher levels of IL-4 (p = 0.0000004), a Th2-type cytokine, IL-17A (p = 0.015), IL-17F (p = 0.023) and IL-22 (p = 0.006), three Th17-type cytokines, compared to peripheral blood T cell clones (Fig. 2). By contrast, IFN-γ production by T cell clones was decreased (p = 0.0001) in the decidua compared to peripheral blood (Fig. 2).

These data confirm the associated production of IL-4, IL-17A, IL-17F and IL-22 by decidual CD4+ T cells in normal pregnancy, thus showing an association between Th2-and Th17-type cytokines by decidual CD4+ T cells in normal pregnancy.

### Prevalence of Th17/Th2 CD4+ cells in the decidua of successful pregnancy whereas of Th17 and Th17/Th1 cells in the decidua of unexplained recurrent abortion

**Fig. 3:**
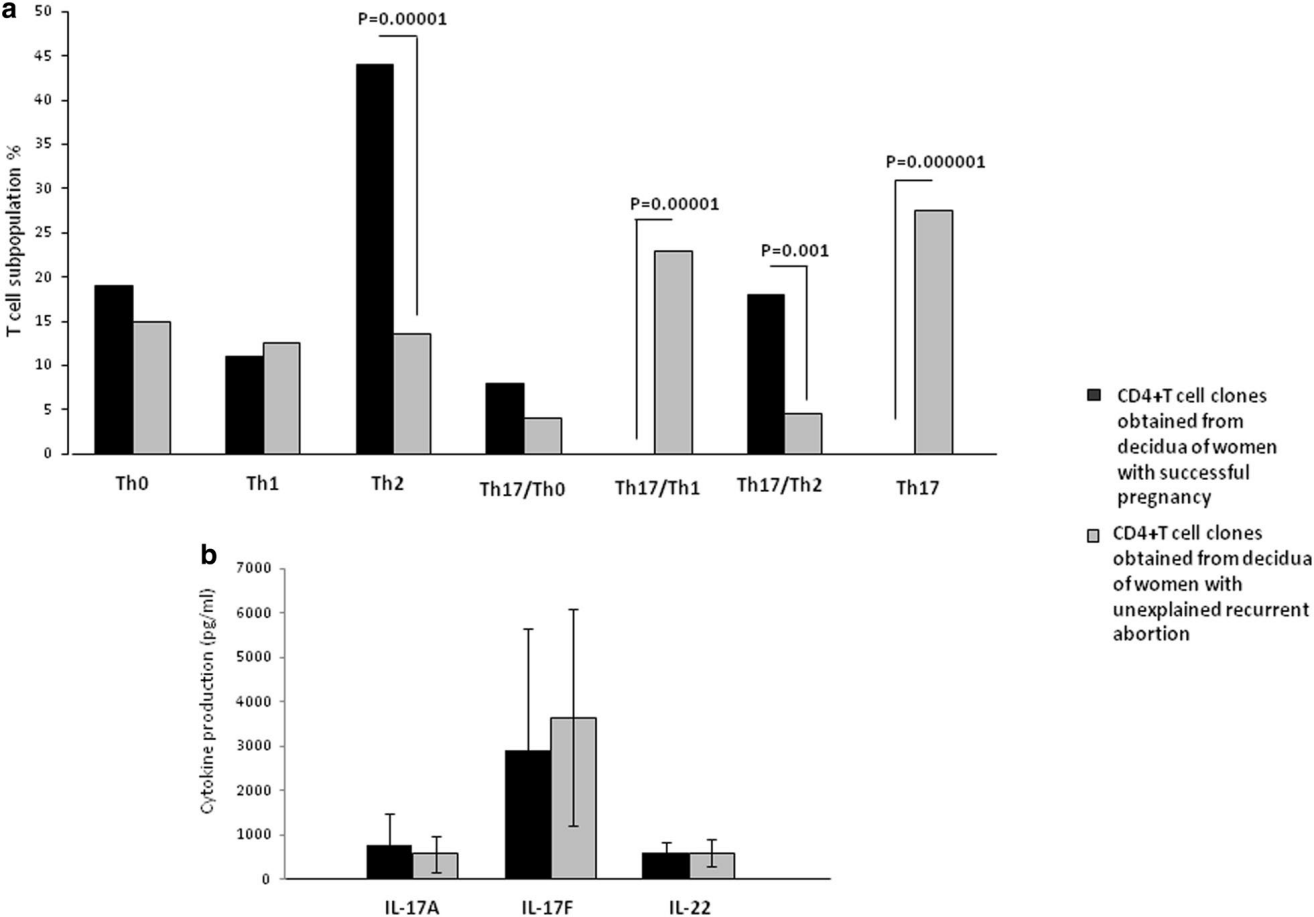
Prevalence of Th17/Th2 cells in the decidua of successful pregnancy but of Th17 and Th17/Th1 cells in the decidua of unexplained recurrent abortion. **a** Analysis of the percentages of Th1-, Th2-, Th0-, Th17-cells and of Th17/Th1, Th17/Th2 and Th17/Th0 in CD4+ T cell clones derived from the decidua of four women with normal pregnancy, and from the decidua obtained from four women suffering from unexplained recurrent abortion. Data are represented as mean ± SEM. The statistical analysis was performed with Chi-square test. **b** 54 and 103 CD4+ T cell clones were generated respectively from the decidua of 4 women with normal pregnancy, and from the decidua obtained from 4 women suffering from unexplained recurrent abortion. IL-17A, IL-17F and IL-22 (mean ± SEM) were measured in the supernatant by multiplex bead-based assay. The statistical analysis was performed with Wilcoxon test

It has been reported that in “inevitable” spontaneous abortion with genital bleeding and in unexplained spontaneous abortion, the number of decidual Th17 cells increased compared to normal pregnancy [31, 33]. Previously, we showed that in normal pregnancy both IL-17A, IL-17F and IL-22 are produced by decidua CD4+ T helper cells in association with IL-4. However, these previous experiments did not show whether the Th17-type cytokines (IL-17A, IL-17F and IL-22) and the Th2-type cytokine, IL-4, are produced by two different decidual CD4+ T cell subsets or if the same CD4+ T cell simultaneously produces the Th17-type and the Th2-type cytokines. In fact, part of human IL-17A-producing cells were found to also produce IL-4 (these cells were named Th17/Th2 [35] and other cells together with IL-17 can produce interferon (IFN)-γ (these cells were named Th17/Th1 [6]. To investigate the possibility that in normal pregnancy the same CD4+ cell subset can produce IL-17 and IL-4, we analyzed not only the percentages of Th1-, Th2-, Th0- and Th17-cells, but also the percentages of Th17/Th1 (producing IL-17A,IL-17F, IL-22 and IFN-γ), Th17/Th2 (producing IL-17A, IL-17F, IL-22 and IL-4) and Th17/Th0 (producing IL-17A, IL-17F, IL-22, IL-4 and IFN-γ). CD4+ T cell clones were derived from the decidua of 4 women with normal pregnancies, who underwent an elective termination of pregnancy, and from the decidua obtained from 4 women suffering from unexplained recurrent abortion (Fig. 3). We found that 26 % of the whole CD4+ T cell clones generated from normal pregnancy produce IL-17 (54/208 T cell clones), whereas 59 % of the whole CD4+ T cell clones generated from unexplained recurrent abortion produce IL-17 (103/174 T cell clones). Thus, according to what was reported by Wang [31] and Nakashima [33], it seems that the percentage of the whole IL-17-producing T cells, without any Th17-type subpopulations analysis, is higher in unexplained recurrent abortion compared to normal pregnancy (p = 0.000001). We found that all the Th17, Th17/Th2 and Th17/Th1 T cell clones produced IL-22. There is no significant difference between the percentage of Th1, Th0 and Th17/Th0 CD4+ T cell clones generated from the decidua of normal pregnancy and those generated from spontaneous abortion (Fig. 3a). In contrast, the percentage of “proper” decidual Th17 (producing only IL-17A, IL-17F and IL-22) (p = 0.000001) and decidual Th17/Th1 (producing IFN-γ plus IL-17A, IL-17F and IL-22) T cell clones (p = 0.00001) were significantly higher in unexplained recurrent abortion compared to normal pregnancy (Fig. 3a). Noteworthy is the observation that no “proper” Th17 and Th17/Th1 T cell clones were ever detected in normal pregnancy decidua. However, the percentages of Th2 (p = 0.00001) and Th17/Th2 (producing IL-4 plus IL-17A, IL-17F and IL-22) (p = 0.001) T cell clones were significantly higher in the decidua of normal pregnancy compared to decidua of women suffering from unexplained recurrent abortion (Fig. 3a).

Measuring the levels of IL-17A and IL-17F produced by the whole IL-17-producing CD4+ T cell clones obtained from normal pregnancy (54 T cell clones) and by the whole IL-17-producing CD4+ T cell clones obtained from unexplained recurrent abortion (103 T cell clones), we did not find a significant increase in IL-17A and IL-17F production by CD4+ T cell clones generated from the decidua from unexplained recurrent abortion compared to the IL-17A and IL-17F production by CD4+ T cell clones generated from the decidua of successful pregnancy (Fig. 3b). Similarly we did not find a significant increase in IL-17A and IL-17F production by CD4+ T cell clones generated from peripheral blood of successful pregnancy and recurrent spontaneous abortions (Additional file 1). These results also indicate that spontaneous recurrent abortions are not necessarily associated with increased levels of IL-17A and IL-17F produced by CD4+ T cells. Moreover, we measured the levels of IL-17 produced by Th17/Th2 T cell clones in normal pregnancy (18 % of T cell clones) and unexplained recurrent abortion (4.5 % of T cell clones), and we found that the levels of IL-17A produced by Th17/Th2 cells in successful pregnancy is higher (9507 ± 7000 pg/ml) compared to the levels of IL-17A produced by Th17/Th2 cells in unexplained recurrent abortion (137 ± 80 pg/ml) (p = 0.0001). These results confirmed that high IL-17 production is not associated with spontaneous recurrent abortion.

We demonstrated that IL-17A, IL-17F, IL-22, together with IL-4, were produced by the same decidual CD4+ T cell subpopulation (the Th17/Th2 cells) in normal pregnancy and not by two different CD4+ T cell subsets. Thus, IL-17 production by decidual CD4+ T cells does not seem to be associated with spontaneous abortion or unexplained spontaneous abortion, as was reported [31, 33]. IL-17 produced by decidual CD4+ T cells, if associated with IL-4 production, is not deleterious for pregnancy outcome.

### Th17/Th2 CD4+ T cells are exclusively present at the implantation site of ectopic pregnancy

Decidual Th17/Th2 cells seem to be important for normal pregnancy development. We wondered whether these cells were present at the implantation site of the embryo and thus could have an important role for embryo implantation. To answer this question, we performed the same kind of cytokine analysis in ectopic tubal pregnancies.

We evaluated not only the percentage of Th1-, Th2-, Th0- and Th17-cells, but also the percentages of Th17/Th1 (producing IL-17A, IL-17F, IL-22 and IFN-γ), Th17/Th2 (producing IL-17A,IL-17F, IL-22 and IL-4) and Th17/Th0 (producing IL-17A, IL-17F, IL-22, IL-4 and IFN-γ) among the CD4+ T cell clones derived from the implantation site of the embryo (N = 133) and those distant from the implantation site in the same fallopian tube (N = 62) of 3 women suffering from ectopic pregnancy.

**Fig. 4:**
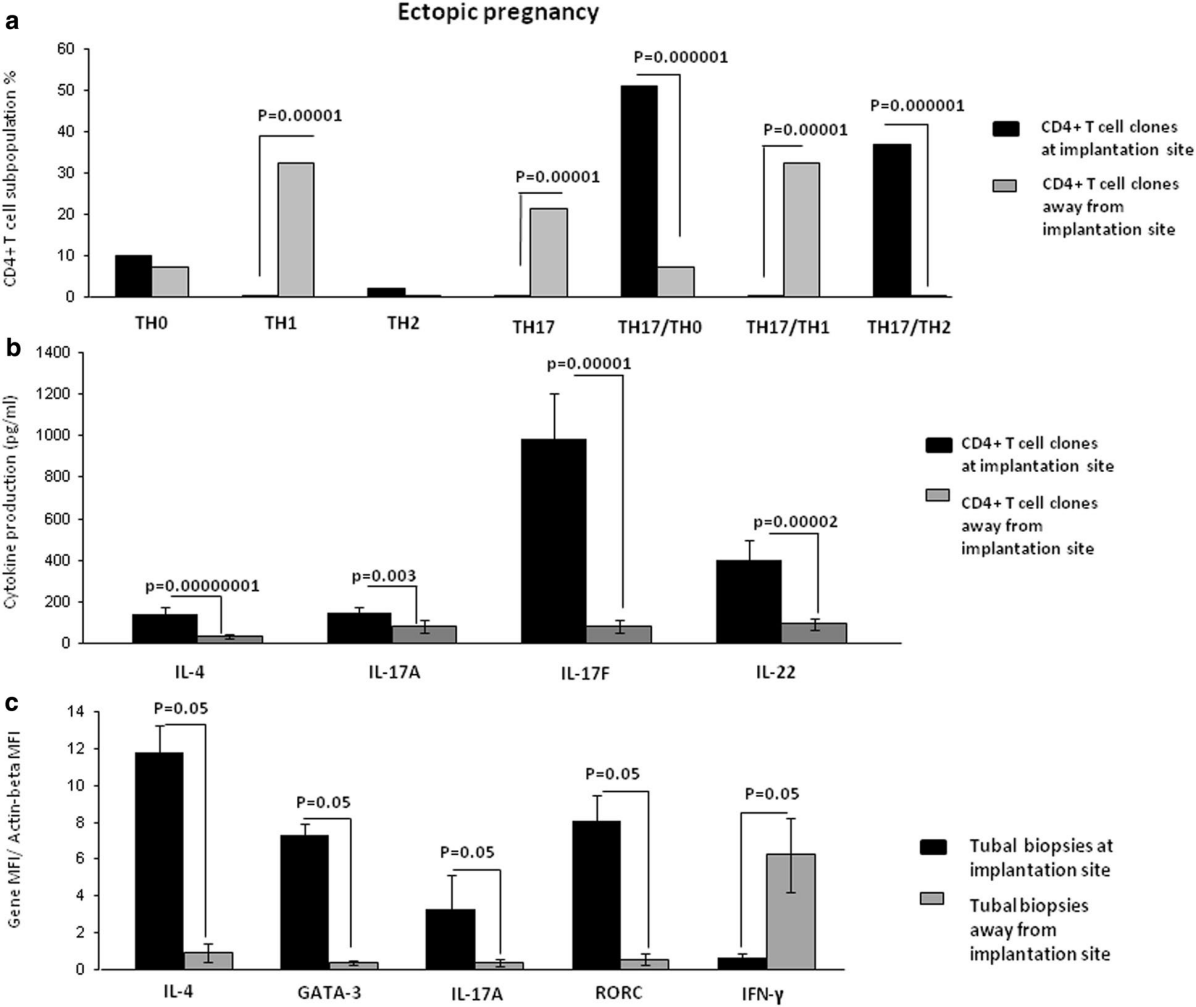
Th17/Th2 cells exclusively found at implantation site of ectopic pregnancy. **a** Percentage of Th1-, Th2-, Th0- and Th17-cells, and of Th17/Th1 and Th17/Th2 and Th17/Th0 among the CD4+ T cell clones derived from the implantation site of embryo (N = 133) and those distant from the implantation site in the same fallopian tube (N = 62) of 3 women suffering from ectopic pregnancy. Data are represented as mean ± SEM. **b** Levels of IL-4, IL-17A, IL-17F and IL-22 produced by the CD4+ T cell clones at the implantation site (N = 133) and distant from the implantation site (N = 62). Data are represented as mean ± SEM. **c** mRNA levels of IL-4, GATA-3, IL-17A, ROR-C and IFN-γ in the biopsies taken at embryo implantation site and distant from the implantation site in the fallopian tube of three women suffering from ectopic pregnancy. For **a**, **c** the statistical analysis was performed with Chi-square test. For **b** the statistical analysis was performed with Wilcoxon test

There is no significant difference in the percentage of pure Th2 and pure Th0 CD4+ T cell clones generated from the implantation site and distant from the implantation site (Fig. 4a). At the implantation site the percentage of Th17/Th2 (p = 0.000001) and of Th17/Th0 (p = 0.000001) CD4+ T cell clones is higher than those clones distant from the implantation site. Conversely, the percentage of Th1(p = 0.00001), pure Th17 (p = 0.00001) and Th17/Th1 (p = 0.000001) is higher apart from the implantation site compared to the embryo implantation site where these 3 types of CD4+ T cells are not present (Fig. 4a). Thus, Th17/Th1 cells are present only outside the implantation site or, as seen above, in decidua of recurrent spontaneous abortions. In other words, it seems that Th17/Th1 and pure Th17 cells, together with Th1 cells, are observed at locations where no implantation of the embryo occurs or when the implantation failed or is not maintained. By contrast, Th17/Th2 are prevalent in normal pregnancy and exclusively present at the implantation site of the embryo.

The levels of IL-4 (p = 0.00000001), IL-17A (p = 0.003), IL-17F (p = 0.00001) and IL-22 (p = 0.00002), produced by the CD4+ T cell clones at the implantation site are higher than the levels of these cytokines distant from the implantation site (Fig. 4b), indicating that there is an increase of production of Th2-type and Th17-type cytokines at the implantation site.

We confirmed these results by determining mRNA expression in Fallopian tube tissue taken at the embryo implantation site and tissue sampled distant from the implantation site of 3 women suffering from ectopic pregnancy (Fig. 4c). At the implantation site, the levels of mRNA for Th2-type molecules (IL-4 and GATA3) and for Th17-type molecules (IL-17A and RORC) were increased compared to the mRNA levels for these molecules distant from the implantation site. In contrast, distant from the implantation site mRNA production of IFN-γ is increased compared to those expressed at the embryo implantation site (Fig. 4c).

### Soluble HLA-G5 mediates the development of Th17/Th2 cells by increasing IL-4 and IL-17A production of the CD4+ T helper cells

We can speculate about the origin of the factor(s) present in the uterine microenvironment that is able to induce Th17/Th2 cells by increasing the production of both IL-4 and IL-17A of the CD4+ T helper cells. Years ago, we reported that progesterone could induce Th2 responses [44]. Recently it has been shown that progesterone inhibits IL-17 production [45]. Thus, progesterone cannot be the factor responsible for Th17/Th2 cell development. Very recently, we showed that soluble HLA-G5 induces an increased production of IL-4 by CD4+ T helper cells [40]. We wondered if HLA-G5 could also induce IL-17A production by the CD4+ T cells and could be the factor responsible for the development of Th17/Th2 cells at the implantation site in normal pregnancy.

To investigate the possible influence of HLA-G5 on IL-4 and IL-17 production of antigen-specific T cells, we generated streptokinase (SK)-specific T cell lines (TCL) from 5 donors cultured in the absence or presence of HLA-G5. As a control, peripheral blood mononuclear cells from the same donors were stimulated with SK in the presence of IL-4, a powerful inducer of Th2 differentiation [46] and IL-12, a potent inducer of Th1 differentiation [47], which indicate that SK-specific TCL are modulated in our culture conditions (data not shown). When we measured the cytokines present in the supernatants of SK-specific T cell lines, we found a significant increase of IL-4 secretion (p = 0.0001) by the SK-specific T cell lines in response to IL-4, and a significant increase of IFN-γ (p = 0.006) in response to IL-12 (data not shown). This suggests that the culture conditions were satisfactory for the modulation of the T cell line cytokine profile.

**Fig. 5:**
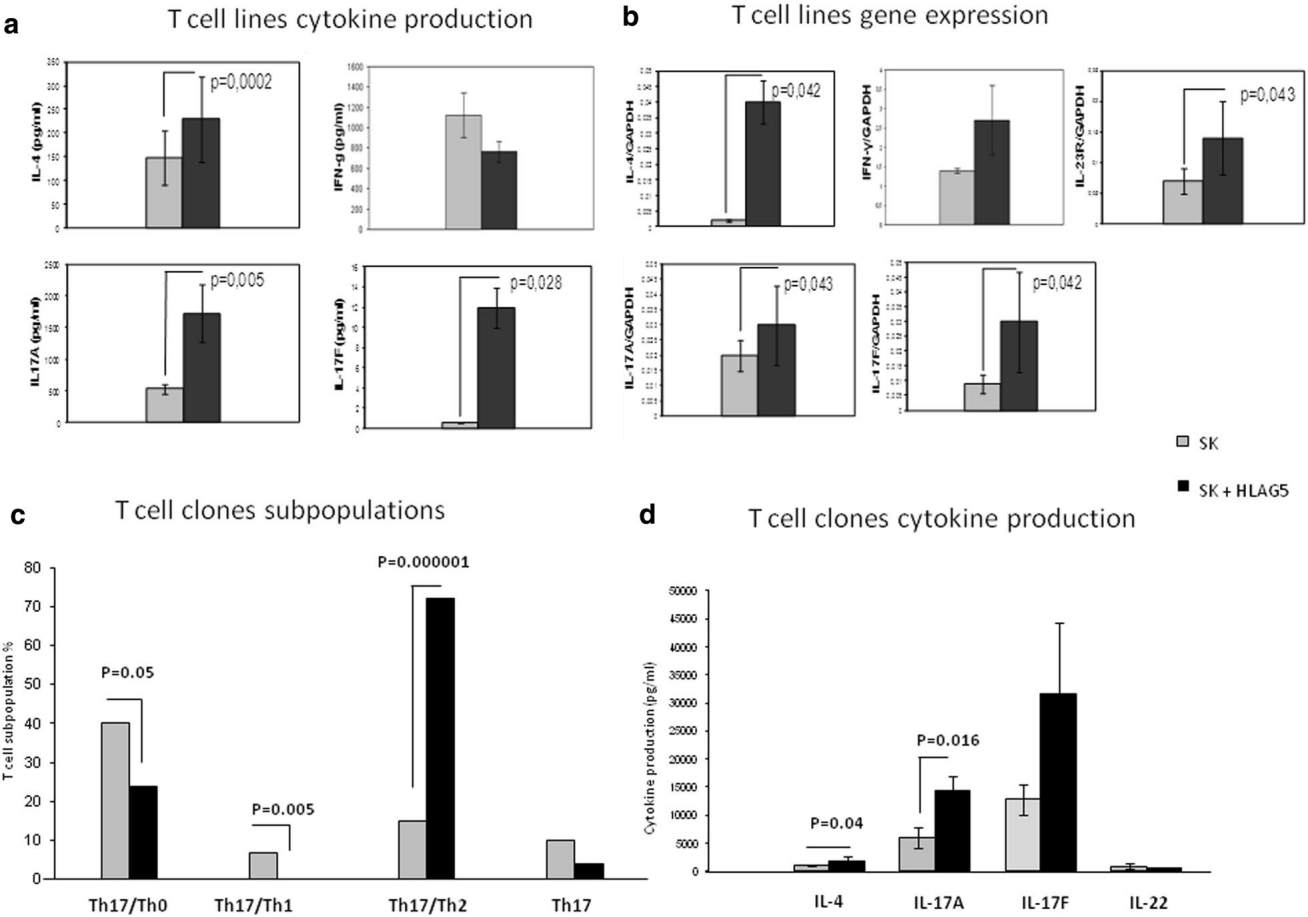
Soluble HLA-G5 mediates the development of Th17/Th2 cells by increasing IL-4 and IL-17A production by the CD4+ T helper cells. **a** IL-4, IL-17A, IL-17F and IFN-γ (mean ± SEM) were measured by bead-based assays in the supernatants of SK-specific T cell lines derived in the presence and in the absence of HLA-G5. **b** mRNA levels (mean ± SEM) of IL-4, IL-17A, IL-17F, IL-23R and IFN-γ were measured by RT-PCR in SK-specific T cell lines derived in the presence and in the absence of HLA-G5. **c** CD4+ T cell clones were derived from the 4 SK-specific T cell lines derived in the presence of HLA-G5 (N = 87) and from the 4 SK-specific T cell lines derived in the absence of HLA-G5 (N = 68) of 4 donors and the levels of IFN-γ, IL-4, IL-22, IL-17A and IL-17F produced by the T cell clones were measured. The percentages of Th17/Th1 and Th17/Th2 and Th17/Th0 and pure Th17 CD4+ T cell clones derived from the SK-TCL modulated in presence or absence of HLA-G5 were analysed. Data are represented as mean ± SEM. **d** The levels of IL-4, IL-17A, IL-17F and IL-22 produced by the Th17/Th2 T cell clones in the SK-specific T cell lines derived in the absence and in the presence of HLA-G5 were measured by bead based assays. Data are represented as mean ± SEM. For **a**, **b** and **d** the statistical analysis was performed with Wilcoxon test. For **c** the statistical analysis was performed with Chi-square test

A statistically significant increase of IL-4 (p = 0.0002), IL-17A (p = 0.005) and IL-17F (p = 0.028) was observed with the SK-specific T cell lines generated in the presence of HLA-G5 1 µg/ml (Fig. 5a). In contrast, IFN-γ production in response to HLA-G5 was not statistically significant (Fig. 5a). We then analyzed the cytokine mRNA levels of SK-specific T cell lines by RT-PCR (Fig. 5b). A statistically significant increase of IL-4 (p = 0.042), IL-17A (p = 0.043), IL-17F (p = 0.042) and IL-23R (receptor expressed by Th17 cells) (p = 0.043) mRNA expression was observed with the SK-specific T cell lines generated in the presence of HLA-G5 compared to the SK-specific T cell lines generated in the absence of HLA-G5 (Fig. 5b). By contrast, no significant differences were observed for IFN-γ mRNA expression between the T cell lines generated in the presence or in the absence of HLA-G5 (Fig. 5b). These findings confirmed the results obtained at the protein level.

We derived CD4+ T cell clones from each of the SK-specific T cell lines of 4 donors (68 T cell clones in the absence of HLA-G5 and 87 T cell clones in the presence of HLA-G5). Subsequent analysis of their ability to produce IFN-γ, IL-4, IL-22, IL-17A and IL-17F (Fig. 5c) was performed. We analyzed the percentages of Th17/Th1 (producing IL-17A, IL-17F, IL-22 and IFN-γ) and Th17/Th2 (producing IL-17A, IL-17F, IL-22 and IL-4) and Th17/Th0 (producing IL-17A, IL-17F, IL-22, IL-4 and IFN-γ) and pure Th17 CD4+ T cell clones derived from the SK-TCL modulated in the presence or absence of HLA-G5 (Fig. 5c, d).

The percentage of Th17/Th2 CD4+ T cell clones increases in the presence of HLA-G5 (p = 0.000001) (Fig. 5c). However, the percentage of Th17/Th1 (p = 0.005) and Th17/Th0 (p = 0.05) T cell clones is decreased in the presence of HLA-G5, but there is no difference in the percentage of Th17 clones in the absence or in the presence of HLA-G5 (Fig. 5c).

The small percentage of Th17/Th2 T cell clones in the absence of HLA-G5 (Fig. 5c), prompted us to analyze the levels of Th2- and Th17-type cytokines produced by the Th17/Th2 T cell clones in the absence and in the presence of HLA-G5. We measured the cytokines present in the supernatants of CD4+ T cell clones derived from the SK-specific T cell lines (Fig. 5d). We found a significant increase in the secretion of IL-4 (p = 0.04) and IL-17A (p = 0.016) by the Th2/Th17 T cell clones derived from SK-TCL generated in the presence to HLA-G 5, compared to Th2/Th17 clones derived from SK-TCL without HLA-G5. By comparison, the levels of IL-17F and IL-22 were not significantly modified (Fig. 5d).

The above findings indicate that HLA-G5 increases the production of both IL-17 and IL-4 by antigen-specific T cells in response to HLA-G5, thus upregulating the development of Th17/Th2 cells found at the site of embryo implantation (Additional file 1: Figure S1).

## Discussion

Our findings seem to confirm the results reported by Wang [31] and Nakashima [33] showing that the percentage of IL-17 producing T cells in the decidua of unexplained recurrent abortion is higher than the percentage of IL-17-producing cells in the decidua of normal pregnancy. However, we did not find a significant increase of the IL-17 production by CD4+ T cell clones generated from the decidua of women with a normal pregnancy compared to the IL-17 production of CD4+ T cell clones generated from the decidua obtained of women suffering from unexplained recurrent abortion. Prior investigations [31, 33] did not make comparative measurements of IL-17 production by decidual CD4+ T cells in normal pregnancy and miscarriages and none identified Th17 subpopulations able to produce IL-4 or IFN-γ together with IL-17 (Th17/Th2 and Th17/Th1, respectively). Moreover, Th17 cells at the implantation site were never investigated. In the present study, we found that the number of IL-17-producing CD4+ T cell clones is increased in the decidua of normal pregnancy compared to peripheral blood, and more importantly, we observed an associated production of IL-4 and IL-17 by a large number of decidual CD4+ T cell clones (18 % of Th17/Th2 clones) in successful pregnancy. Accordingly, we found that freshly purified CD4+ T cells obtained from decidua of elective terminations of pregnancy, which have already been activated by trophoblast in vivo as recently demonstrated [40], can produce spontaneously and simultaneously IL-17 and IL-4 without any additional in vitro stimulation. The association of IL-17 and IL-4 production has been observed in allergic disorders known to be characterized by a Th2-type response [35], but the percentage of Th17/Th2 cells in peripheral blood of healthy subjects is very low (0.04 %) and increases only slightly in the peripheral blood of allergic patients with chronic asthma (1.3 %). To our knowledge, our study demonstrates for the first time a physiologic condition, namely successful pregnancy, in which the percentage of Th17/Th2 cells is markedly elevated. Interestingly, it was reported that in allergic disorders 0 % of Th17/Th2 T cell clones derived from purified peripheral blood CD161+CCR6+Th17 cells were obtained, but if CD161+CCR6+Th17 cells were cultured in the presence of IL-4, the percentage of Th17/Th2 cells increased to 14 % [35]. These findings suggest that Th17 cells switch toward Th17/Th2 cells, which could be modulated by the strongly Th2-type microenvironment induced in particular by high concentrations of progesterone in the decidua [44]. Thus, our findings contradict those reported by other authors [31, 32] who suggested that Th17 cells may promote miscarriage and be deleterious for pregnancy because of their capacity to induce alloantigens rejection [13]. Our data do not support a pathogenic role for IL-17 producing CD4+ T cells in pregnancy, rather we suggest that a beneficial role of Th17 cells play a beneficial role when they also produce IL-4 during the first trimester of human pregnancy. Furthermore, the Th17/Th2 cells are present only at the embryo implantation site. By contrast, Th17/Th1 cells and Th17 cells are prevalent, not only when the implantation fails but also when embryo implantation does not occur. Th17/Th1 could be involved as a potential cause of the abortion or could just be a consequence of this event. The functional activity of Th17/Th1 cells may be the result rather than the cause of pregnancy failure. Even in this case, however, the production of IFN-γ and IL-17, known to be involved in allograft rejection, may aggravate the situation and accelerate fetal allograft rejection and thus spontaneous abortion.

We have also demonstrated that HLA-G5, a factor present in the pregnant uterus microenvironment [48] and produced by both trophoblast and embryo, is responsible, at least in part, for the development of Th17/Th2 cells which in turn seem to be crucial for successful embryo implantation.

## Conclusion

Infection-related immunity during gestation, responsible for a large number of miscarriages, seems to be preferentially directed towards combating extracellular microbial pathogens. During fetal development, interleukin (IL)-23, IL-10 and IL-6, as well as T-helper-17 (Th17)-mediated immune responses, are upregulated, whereas tumour necrosis factor-α (TNF-α) and IL-1β- and Th1-mediated immune responses are downregulated in the intrauterine environment (in both the fetal compartment and amniotic compartment) [49]. We hypothesize that IL-17/IL-4 producing decidual CD4+ T cells could be beneficial and useful for the maintenance of pregnancy, because they may promote an adequate response required to protect the mother against dangerous extracellular pathogens. In addition, the IL-4 produced by these cells together with the Th2 cells in the decidua [22] may induce tolerance towards paternal HLA-C expressed by the conceptus, through the production of IL-4. Moreover, IL-17 could be beneficial for successful pregnancy because it could promote the proliferation and invasion of human extravillous cytotrophoblast [50], important for gestational development.

We postulate that IL-17 could be essential for the success of pregnancy at certain stages of pregnancy but not so important or deleterious at other stages. The chronology of action of IL-17, alone or in association with other cytokines, should be further investigated to better understand the mechanisms by which pregnancy may or may not be affected by Th17 cells.

## Additional file

10.1186/s12948-016-0039-y IL-4, IL-17A and IL-17F production by peripheral blood CD4+T cell clones in successful pregnancy and unexplained spontaneous abortion.Levels of IL-4, IL-17A and IL-17F produced by the CD4+ T cell clones (N=40) obtained from the peripheral blood of women who underwent elective abortion and from women who underwent spontaneous abortion (N=40) were measured by multiplex bead-based assay. Data are represented as mean ± SEM ( pg/ml) and the statistical analysis was performed with Wilcoxon test.
