# Supplementary figures and images for: Interleukin-17-producing decidual CD4+ T cells are not deleterious for human pregnancy when they also produce interleukin-4
Source: Clin Mol Allergy. 2016 Jan 21;14:1. doi: 10.1186/s12948-016-0039-y (PMC4721137; doi:10.1186/s12948-016-0039-y)

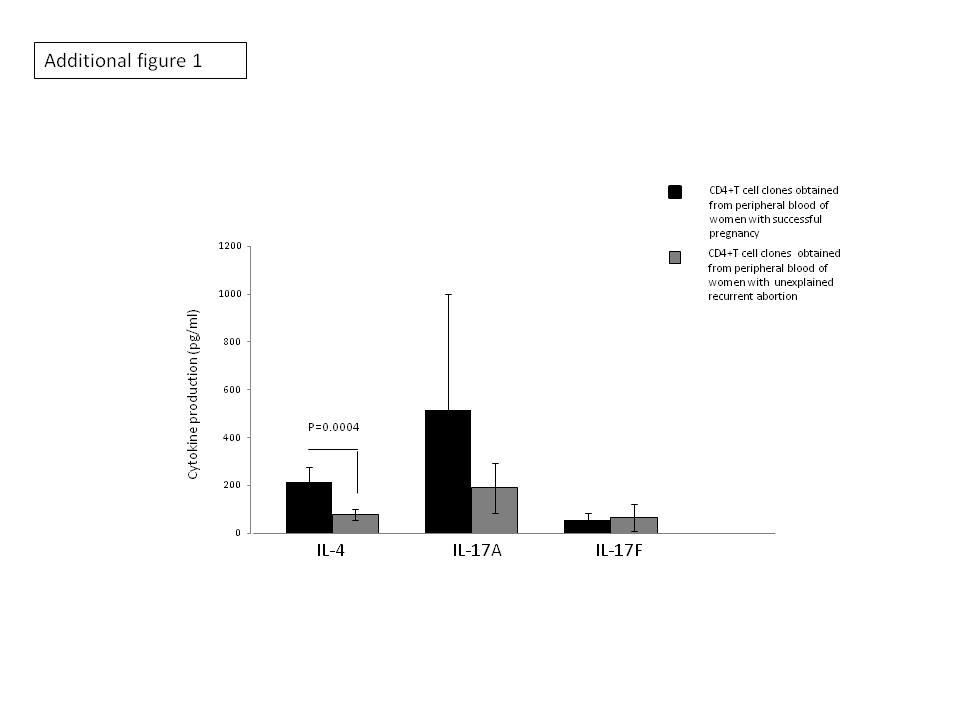

Supplement: Supplementary file 1 — 10.1186/s12948-016-0039-y IL-4, IL-17A and IL-17F production by peripheral blood CD4+T cell clones in successful pregnancy and unexplained spontaneous abortion.Levels of IL-4, IL-17A and IL-17F produced by the CD4+ T cell clones (N=40) obtained from the peripheral blood of women who underwent elective abortion and from women who underwent spontaneous abortion (N=40) were measured by multiplex bead-based assay. Data are represented as mean ± SEM ( pg/ml) and the statistical analysis was performed with Wilcoxon test. [file 12948_2016_39_MOESM1_ESM.tiff]
